# Effect of temperature stress on the early vegetative development of *Brassica oleracea* L.

**DOI:** 10.1186/s12870-015-0535-0

**Published:** 2015-06-16

**Authors:** Víctor M. Rodríguez, Pilar Soengas, Virginia Alonso-Villaverde, Tamara Sotelo, María E. Cartea, Pablo Velasco

**Affiliations:** Group of Genetics, Breeding and Biochemistry of Brassicas. Misión Biológica de Galicia (MBG-CSIC), Apartado 28, 36080 Pontevedra, Spain; Group of Viticulture, Misión Biológica de Galicia (MBG-CSIC), Apartado 28, 36080 Pontevedra, Spain

**Keywords:** *Brassica*, Physiology, Photosynthesis, Thermal stress

## Abstract

**Background:**

Due to its biennual life cycle *Brassica oleracea* is especially exposed to seasonal changes in temperature that could limit its growth and fitness. Thermal stress could limit plant growth, leaf development and photosynthesis. We evaluated the performance of two local populations of *B. oleracea*: one population of cabbage (*B. oleracea capitata* group) and one population of kale (*B. oleracea acephala* group) under limiting low and high temperatures.

**Results:**

There were differences between crops and how they responded to high and low temperature stress. Low temperatures especially affect photosynthesis and fresh weight. Stomatal conductance and the leaf water content were dramatically reduced and plants produce smaller and thicker leaves. Under high temperatures there was a reduction of the weight that could be associated to a general impairment of the photosynthetic activity.

**Conclusions:**

Although high temperatures significantly reduced the dry weight of seedlings, in general terms, low temperature had a higher impact in *B. oleracea* physiology than high temperature. Interestingly, our results suggest that the *capitata* population is less sensitive to changes in air temperature than the *acephala* population.

**Electronic supplementary material:**

The online version of this article (doi:10.1186/s12870-015-0535-0) contains supplementary material, which is available to authorized users.

## Background

Due to their sessile lifestyle plants are especially exposed to environmental changes that modulate their growth and development. Optimal plant growth takes place within more or less strict environmental conditions. Outside this optimal range, plants suffer stresses which limit their growth and productivity. In agriculture some of these abiotic stresses can be minimized by using irrigation and fertilization. Other stresses, however, are difficult to overcome and fluctuations in air temperature are a clear example. Variations in temperature are one of the principal factors that drive plant phenology. Stratification and vernalization are well known physiological processes that are triggered by transient exposure to low temperatures [1, 2]. Seasonal changes in temperature also promote many developmental processes (i.e. flowering, germination or grain filling) [3, 4]. However, above or below certain thresholds, temperatures limit geographical distribution and productivity of many important crops.

Contrary to metazoans, plants do not have specialized cell types that allow perception of temperature fluctuations. The mechanisms through which plants perceive temperature has been proposed to be similar for both, high and low temperatures, although the intracellular signaling and physiological response differ between both stimuli [5]. The first structure that responds to temperature fluctuations is the plasma membrane. Both, high and low temperatures, cause changes in the fluidity of this structure which activates an intracellular signal cascade [6]. Associated with the plasma membrane, the cytoskeleton is another sensor of temperature fluctuations. Exposure of plants to growth-limiting temperatures induces the depolymerization of microtubules and microfilaments [7, 8]. These two structures are intimately involved in cell morphogenesis [9] and its rearrangement may explain variations of the leaf shape in plants growth under extreme temperatures [10].

Probably, the cellular component most sensitive to temperature fluctuations is the photosynthetic apparatus. The primary targets of thermal stress on the photosynthetic apparatus in plants are the photosystem II (PSII) and the carbon fixation by Rubisco [11]. An early effect of temperature in the photosynthetic apparatus is the inhibition of the activity of the PSII. This phenomenon has been broadly studied in the last decade to the extent that the chlorophyll fluorescence analysis is nowadays an experimental approach routinely used in physiological studies [12, 13]. When illuminated, the antennae within the photosynthetic membrane absorb energy that is transferred to the reaction center. The fraction of energy that could not be trapped by the reaction center is then dissipated as heat or fluorescence. The amount of emitted fluorescence could be easily measured through fluorescence spectroscopy. Upon illumination the fluorescence emission is not constant but exhibits a fast rise followed by a decline to reach a steady level [14]. When fluorescence values recorded during the fast rise are plotted against time on a logarithmic scale (OJIP curve), different phases became visible [15]. Based on the appearance of these phases Strasser et al. [16] have developed a concept that tries to describe and explain changes in the rise kinetics and amplitudes of these phases in response to stress conditions. Based on this concept, equations to calculate a set of parameters were derived (the so-called JIP-test).

Crops with a biennial life-cycle are exposed to seasonal temperature variations ranging from below zero to more than 40 °C. Among biennial plants, *Brassica oleracea* L. stands out as one of the most important species in the world from an economical point of view. Human selection has led to the development of a range of cultivars within this species in which different organs are used for human or livestock consumption [17]. Originally domesticated in Atlantic coastal regions of Europe, cultivars of this species are nowadays cultivated worldwide and grown under a wide range of climate conditions. This wide diversity may be reflected in different mechanisms to respond to thermal stress among the different cultivars of this species. Recently, we have performed a study to elucidate the impact of high temperatures on a broad set of local populations of *B. oleracea* during early development [18]. The variability observed in this analysis prompted us to perform a more detailed analysis of the effect of thermal stress on the early development of *B. oleracea*. Therefore, the goal of the present investigation was to analyze the physiological response of two cultivars of *B. oleracea* grown under extreme temperatures mainly focused on the effect of stressful temperatures on the performance of the photosynthetic apparatus and leaf growth.

## Results and discussion

We studied the effect of stressful temperatures on the early development of *B. oleracea*. Based on a previous evaluation, we selected two populations of *B. oleracea* (one cabbage and one kale population) that showed a good early development under heat conditions [18]. These two cultivars were also selected because they have a common origin (Northwestern Spain) and show similar seasonality. Preliminary evaluations allow us to stablish the limiting temperatures to carry out further evaluations (constant 12 °C for chilling experiments and constant 32 °C for heat experiments). Below 12 °C seedlings were unable to germinate and above 32 °C leaf expansion was dramatically compromised.

**Fig. 1:**
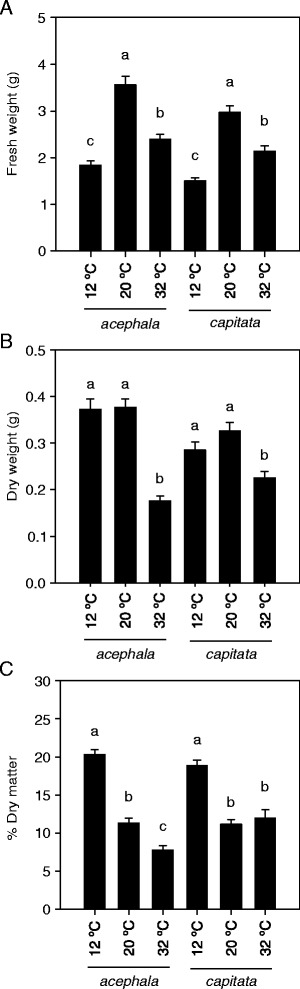
Effect of thermal stress on biomass production of two cultivars of *Brassica oleracea* exposed to chilling (12 °C) and high (32 °C) temperatures. **a**. Fresh weight (g) of the aerial part of seedlings grown under cold and heat conditions. **b**. Dry weight (g) of the aerial part recorded after drying in a oven at 80 °C until constant weight. **c**. Percentage of dry weight. In all pannels bars denote the mean of 20 measurements ± SE. Mean values within each cultivar with different letters are signifficantly different (*P* < 0.05)

As expected, thermal stress produces a significant reduction of the fresh weight of the aerial part of both varieties (Fig. 1a). This reduction was especially marked when plants were grown under chilling conditions, since under such conditions fresh weight was reduced by 50 % compared to values observed under control temperature (20 °C) (Fig. 1a). Curiously, plants grown under chilling conditions did not show a reduction of the dry weight compared to data obtained under control conditions (Fig. 1b), although they showed a significantly higher percentage of dry matter than plants grown under control or high temperature conditions (Fig. 1c), indicating a significant reduction of the leaf water content. Previous studies have reported that plants exposed to cold perform in a similar way as plants exposed to drought, concerning water content [19]. Our experiments were performed with excess of irrigation to remove the effect of drought from the physiological response, which could explain why there is not reduction of the water content in plants exposed to high temperature conditions. Similar results were previously reported in *Nicotiana tabacum* [20]. Although, the mechanism by which cold temperatures influence the hydric status of the plant is unclear, our results suggest that, at least under our experimental conditions, these are independent of those observed under high temperatures and also independent of the water available.

**Fig. 2:**
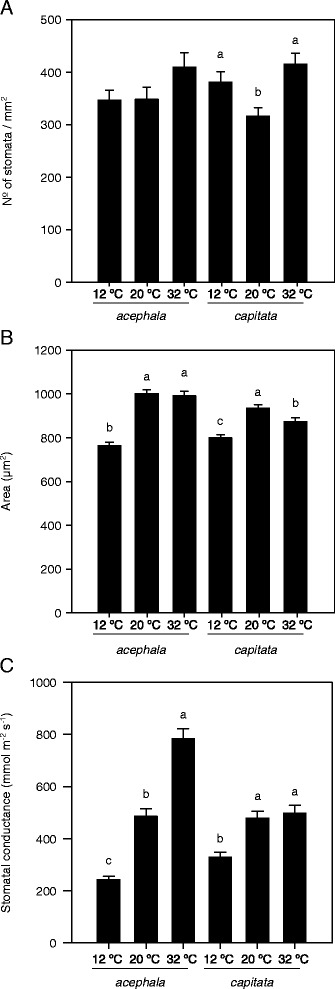
Incidence of temperature on anatomical and functional characteristics of stomata in two cultivars of *Brassica oleracea* exposed to chilling (12 °C) and high (32 °C) temperatures*.* **a**. Number of stomata per mm^2^. **b**. Area of stomata (μm). **c**. Stomatal conductance measured with a leaf porometer. In all panels bars denote the mean of at least 20 measurements ± SE. Mean values within each cultivar with different letters are signifficantly different (*P* < 0.05)

The leaf water content is the result of the equilibrium between water absorption and evapotranspiration. Water absorption through the roots is promoted by increasing temperatures as well as the movement of water within the plant that has been attributed to changes in membrane fluidity and permeability, changes in water viscosity or a combination of both [21–23]. Likewise, the hydraulic conductance of the plant changes linearly with temperature and stomata can directly respond to variations in this parameter by increasing transpiration [22, 24]. For this reason we measured different parameters related to stomata anatomy and functionality. In our experiment, the number of stomata per mm^2^ was not significantly affected by the temperature in the *acephala* group, whereas in the *capitata* group an increase was observed in both stress conditions (Fig. 2a). The size of these stomata was affected by temperature in both groups. Smaller stomata were observed under chilling conditions compared to the size observed under control conditions (Fig. 2b). In the case of the *capitata* group there was also a reduction of the size of the stomata under heat conditions compared to the size observed under control conditions. However, the stomatal-related trait most affected by temperature was the stomatal conductance. For both groups the lowest conductance was recorded under chilling conditions (Fig. 2c). Interestingly, under heat conditions, the stomatal conductance of the *acephala* group significantly increases compared to control conditions whereas the percentage of dry matter decreases when temperature increases (Fig. 1b, Fig. 2c), in concordance with previous theories exposed by [22, 23]. However, in the case of the *capitata* group there are no differences between percentage of dry matter and stomatal conductance between 20 and 32 °C, suggesting that plants from the *capitata* group are more tolerant to high temperatures.

**Fig. 3:**
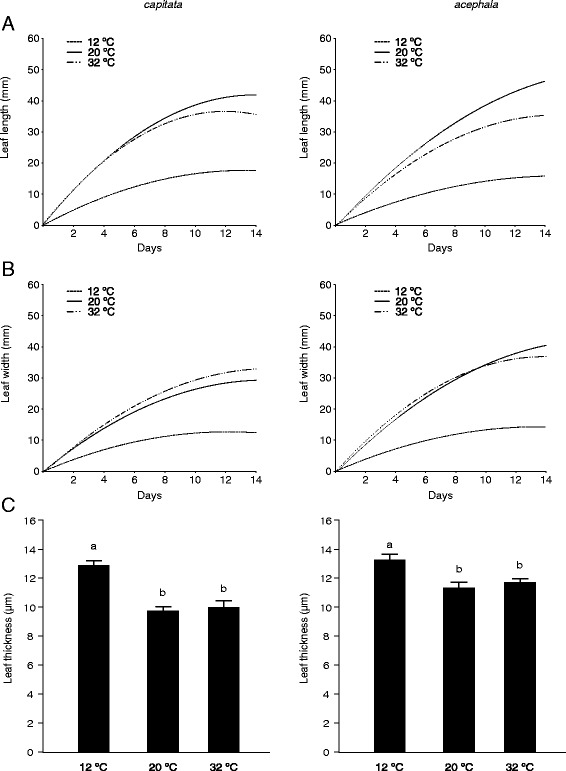
Effect of temperature on leaf size and thickness of two cultivars of *Brassica oleracea*. Leaf length (**a**) and width (**b**) were recorded every other day during 14 days on the second leaf of 30 plants. The curves are quadratic functions fit to the data. **c**. Mean ± SE of leaf thickness recorded with a dial indicator. Mean values within each cultivar with different letters are signifficantly different (*P* < 0.05)

Low temperatures also modulate leaf growth. Plants grown under chilling conditions developed smaller leaves than those grown under control or heat conditions (Fig. 3a and b). Growth under low temperatures often results in significant alterations in leaf morphology. The most noticeable effect is a reduction in specific leaf area (the ratio of leaf area to leaf dry mass) [25]. It is also remarkable that under cold conditions the leaves become thicker than those observed under control or high temperature (Fig. 3c). It has been previously reported that plants grown under chilling conditions show reduced leaf expansion and increased mesophyll thickness [26, 27].

Since temperature affects leaf size, we wondered whether it could affect also the leaf shape. Juvenile leaves of *B. oleracea* have an oval shape. Although the leaves of both varieties follow this general rule, under control conditions the *acephala* group developed leaves slightly longer than its width, whereas the opposite behavior was observed in the leaves of the *capitata* group (see Additional file 1: Figure S1). There was no effect of temperature in the leaf shape of the *capitata* group, whereas the leaves of the *acephala* group developed under thermal stress become longer than its width (see Additional file 1: Figure S1).

**Fig. 4:**
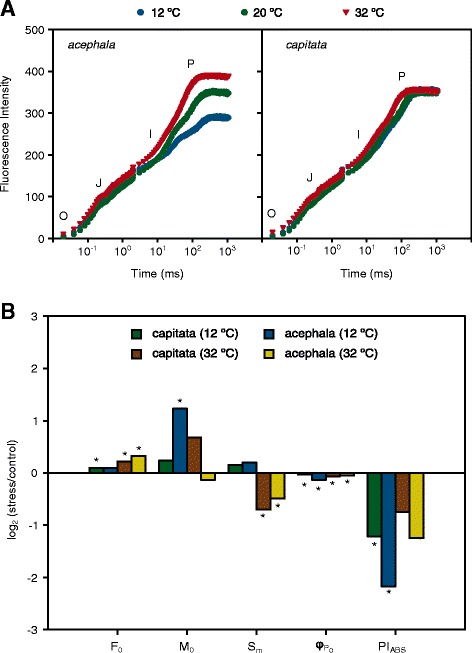
Effect of temperature on the fluorescence transient and JIP test parameters of two cultivars of *Brassica oleracea* expossed to chilling (12 °C) and high (32 °C) temperatures*.* **a**. Chlorophyll *a* fluorescence transient curve expressed on a logaritmic time scale. **b**. Different parameters (F_0,_ minimal fluorescence; M_0_, initial slope of the fluorescence transient; S_m_, area above the OJIP transient; φ_PO,_ maximum quantum yield of primary photochemistry; PI, performance index of fast fluorescence transient in two cultivars of *Brassica oleracea*. Data from the control were used to normalized the different parameters. Log2-transformed normalized values are represented on ordinate. * *P* < 0.05 (treatment *vs.* control)

At the autotrophic stage plant growth strongly depends on the capacity of the photosynthetic apparatus to fix carbon. Photosynthesis is one of the most affected cellular reactions by environmental changes; concretely the PSII activity is especially sensitive to thermal stress [5]. An indirect approximation to the activity of the PSII could be easily measured by using a portable fluorimeter. To determine which of the different stages of the electronic transport could be affected by thermal stress we plotted the fluorescence kinetics in a logarithmic time scale to obtain the so-called OJIP transient curve. The different steps that arise have been associated to different redox states of the components of the electron transport chain [28]. In our experiment, the fluorescence pattern during the first second after transient illumination was similar between the *acephala* and *capitata* groups grown under control conditions (Fig. 4a).

According to experimental data the OJIP transient curve could be divided in two mechanistic phases, the “photochemical phase” (O-J rise) and the “thermal phase” (J-I-P rise) [29]. Thermal stress induces a significant increase of fluorescence at the O step in the *B. oleracea* seedlings which is more prominent under heat than under chilling conditions (see Additional file 2: Figure S2). Under optimal conditions the O step represents the minimal fluorescence intensity (F_0_) [30]. The F_0_ represents the fluorescence emission when all the primary quinone-type acceptors (Q_A_) of the reaction center are in the oxidized state. We observed a significant increase of the F_0_, especially when seedlings were grown under high temperatures. Such an increase has been observed previously in other crops [31, 32], and it has been associated to a dissociation of part of the outer antenna from the rest of the PSII [33] or to a shift in the equilibrium between the electron acceptors Q_A_ and Q_B_ which enhance back electron transfer from Q_B_ to Q_A_ [31, 34]. In this later scenario, Q_A_ will remain partially reduced in darkness and the O-step no longer represents the F_0_.

Nevertheless, the fluorescence kinetics during the “photochemical phase” was similar to that observed under control conditions, indicating that the rate of Q_A_ reduction during early photochemistry was not significantly affected by temperature [35]. This result was confirmed by quantifying the velocity of fluorescence rising during the first milliseconds following a dark to light transition which could be determined by the initial slope of fluorescence (M_0_). This parameter was not significantly affected by temperature except for the *acephala* group under chilling conditions that showed an increase of the initial fluorescence (Fig. 4b).

Several authors reported a new transient step (named K-step) in data obtained from plants cultivated under stress when the OJIP curve is represented as the kinetics of relative variable fluorescence (Vt) in a logarithmic time scale, especially under heat stress [33, 36–38]. This step has been associated with damage to the donor side of PSII by thermal stress [35]. We did not observe an obvious K-step under our experimental conditions (data not shown).

The pattern of fluorescence transient varied among temperatures and cultivars in the “thermal phase” (Fig. 4a). A tendency to increase fluorescence values under high temperature was observed for both genotypes; whereas the opposite was observed under cold temperatures (see Additional file 2: Figure S2). The magnitude of such variation differed between cultivars, being the variation recorded on the *capitata* group less pronounced than that recorded on the *acephala* group. Beyond the differences in amplitude there were also differences in the rise kinetics that can be related to stoichiometric differences in the composition of the photosynthetic electron transport chain [39].

Contrary to the “photochemical phase” there is a controversy in the literature about the molecular mechanisms behind the fluorescence kinetics at the “thermal phase” (for review see [29]). Duysens and Sweers [40] postulated in 1963 that the fluorescence changes reflect primarily changes in the redox state of Q_A_, in a way that the maximum fluorescence is reach when the pool of Q_A_ is completely reduced [29]. However, in the last decades, alternative models have been proposed that implies the involvement of second processes influencing the fluorescence rise [41]. Strasser et al. [42] carried out a simulation with three possible scenarios, considering a pure Q_A_ model or the influence of an alternative quencher and they concluded that all these models fitted satisfactorily with the results. Since the JIP-test is based in a pure Q_A_ model we interpreted our results based on this model, keeping in mind that alternative explanations may be considered.

An important parameter that influences the fluorescence kinetics is the multiple turn-over of Q_A_ that is correlated to the area above the OJIP curve (S_m_) [16]. In our experiment, this area was not affected by chilling temperatures but significantly decreased when both varieties were exposed to high temperatures, indicating that fewer electron acceptors are available in the electron transport chain. However, due to the fact that both genotypes perform similarly, the Q_A_ turn-over does not explain the differences observed in the fluorescence kinetics during the “thermal phase”.

The overall performance of the activity of the PSII was estimated using the maximum quantum yield of primary photochemistry (φ_P0_) which is equivalent to the Fv/Fm parameter [30]. Under chilling and heat conditions, this parameter was significantly reduced compared to values observed under control conditions, indicating that the PSII undergoes physiological changes due to thermal stress (Fig. 4b).

**Fig. 5:**
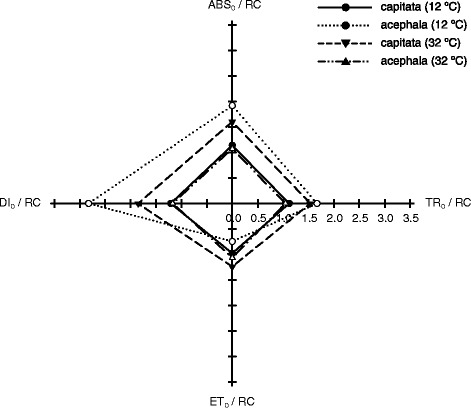
Spider plot representation of specific fluxes per reaction center in two cultivars of Brassica oleracea expossed to control (20 °C), chilling (12 °C) and high (32 °C) temperatures. Energy fluxes are expressed based on the theoretical number of reaction centers (RC). Absorption per RC (ABS/RC); electron transport (ET_0_/RC); trapping (TR_0_/RC); dissipation (DI_0_/RC)

The photosynthetic energy flux may be divided in four different steps. These steps represent the photon flux absorbed by the antenna pigments and creating excited chlorophyll (ABS), the excitation energy that is channeled as trapping flux (TR), the excitation energy that creates an electron transport that leads to CO_2_ fixation (ET) and the energy that is dissipated as heat or fluorescence (DI) and are normalized by reaction center (RC). The performance of both varieties in these four steps was significantly different (Fig. 5). Chilling temperatures have a major impact increasing the energy absorbed (ABS/RC) and trapped (TR/RC) in the *acephala* group, whereas the electron transport flux (ET/RC) decreased. A concomitant increase in the dissipation flux (DI/RC) was observed, indicating that the efficiency of the photosynthesis was reduced. However, high temperatures have little impact in these fluxes in this group and just a slightly increase of the dissipated flux was observed.

The opposite behavior was observed in the *capitata* group. The performance of this group under chilling conditions was similar to that observed for the *acephala* group at heat conditions. However, under heat conditions *acephala* group showed an increase of the four steps studied, indicating that most energy is transmitted through the photosynthetic apparatus.

## Conclusions

Our results suggest that the *capitata* population is less sensitive to changes in air temperature than the *acephala* group; low temperature has a high impact in *B. oleracea* physiology, especially on photosynthesis and fresh weight, although there was not effect on dry weight. Under high temperatures there was a reduction of the fresh weight that could be associated to a general impairment of the photosynthetic activity.

## Methods

### Plant material and growth conditions

One population of cabbage (*B. oleracea capitata* group) and one population of kale (*B. oleracea acephala* group) were obtained from the *Brassica* seed bank of the Misión Biológica de Galicia (CSIC-Spain). Seeds were planted in multi-pot trays filled with sterilized peat (Gramoflor GmbH & Co. KG, Vechta, Germany) with one seed per cavity. Seedlings were grown under fluorescent light (228 μmol m^−2^ s^−1^) in a 14 h light/10 h dark light regime and watered as needed. A constant day/night temperature regime was set up at 20 ± 1 °C for control conditions. The thresholds of high and low temperatures were established experimentally. Heat experiment was performed at 32 ± 1 °C, since above this temperature seedling growth was dramatically reduced and leaf expansion compromised. Chilling experiment was established at 12 ± 1 °C, since lower temperatures reduced dramatically seed germination and seedling survival.

### Morphometric analysis

Leaf growth rate was determined by measuring the maximal length (maximal length from the apical to the basal part of the leaf) and width (measured at the leaf mid-point) of the second leaf of 30 plants every other day until the 14th day after the first data was recorded. Measurements were recorded using a digital caliper (Metrica, Barcelona, Spain). Leaf thickness was measured in 20 plants at the end of the experiment with an AMES 212.1 dial indicator (B.C. AMES CO., Waltham, MA, USA).

### Physiological parameters

Chlorophyll *a* fluorescence was recorded in the second leaf of 20 plants from each population at the V4 developmental stage. Fluorescence was measured with a portable fluorometer (OS-30p Chlorophyll Fluorometer, OptiScience, Inc., Hudson, NH USA) and recorded up to 1 s with a data acquisition rate of 100 readings ms^−1^ for the first 2 ms and 1 reading ms^−1^ thereafter. Fluorescence transient was induced by red light of 3000 μmol m^−2^ s^−1^ provided by an array of 3 light-emitting diodes (peak at 660 nm) using plants dark adapted for 1 hour. Fluorescence data were analyzed according to the JIP test (see Additional file 3: Table S1) [16, 43].

Stomatal conductance was recorded using a SC-1 leaf porometer (Decagon Devices Inc., Pullman, WA, USA) in the second leaf of 20 plants per population and temperature at V4 developmental stage.

### Stomatal measurement

Leaf printing was carried out following Chen et al. [44] with a few modifications. Briefly, a leaf print (approx. size 1 × 1 cm) was obtained from the base of the second leaf from 15 plants in a V4 developmental stage per population and temperature with transparent nail polish from the abaxial leaf lamina close to the principal nerve. Observations were made on a Nikon Eclipse E200 light microscope and the number of stomata per visual field (0.196 mm^2^) was recorded for each sample. Images were captured using a Nikon DS-F11 camera under bright field and the width and length of 15 stomata per plant of each population and temperature were measured using the ImageJ Software [45].

### Statistical analysis

Analyses of variance were performed for each population using the procedure GLM of SAS [46] using temperatures as the classification variables. Temperature was considered as fixed. Comparisons of means were made by using the Fishers’ protected LSD at *P* = 0.05.

## Additional files

Additional file 1: Figure S1. Leaf growth parameters under thermal stress. Graph representation of leaf growth parameters of two populations of *Brassica oleracea* under thermal stress conditions. Simple linear regression curves of each temperature are represented. Additional file 2: Figure S2. Normalized chlorophyll a fluorescence transient curve. Chlorophyll a fluorescence transient curve. Data from the control were used to normalize the curve. Additional file 3: Table S1. List of parameters of the JIP-test. Summary of parameters and formula description using data extracted from the OJIP transient.

## Abbreviations

PSII: Photosystem II
Q_A_: Primary quinone-type acceptor
Q_B_: Secondary quinone-type acceptor
ABS/RC: Absortion flux per reaction center
TR/RC: Trapped energy flux per reaction center
ET/RC: Electron transport flux per reaction center
DI/RC: Dissipated energy flux per reaction center
