# Supplementary figures and images for: Effect of temperature stress on the early vegetative development of Brassica oleracea L
Source: BMC Plant Biol. 2015 Jun 16;15:145. doi: 10.1186/s12870-015-0535-0 (PMC4467057; doi:10.1186/s12870-015-0535-0)

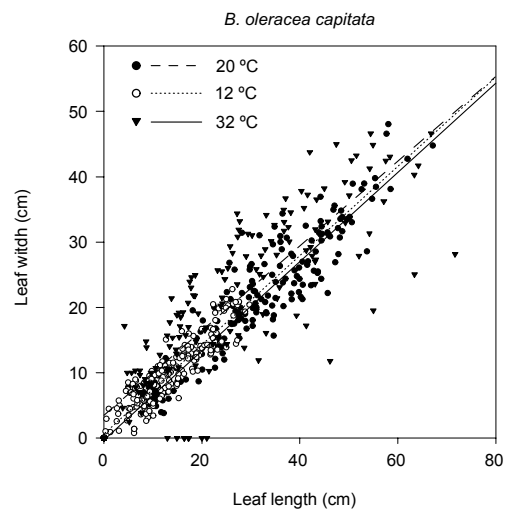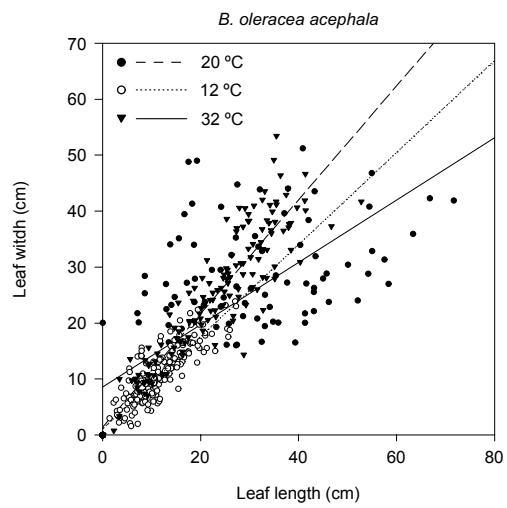

Supplement: Additional file 1: Figure S1. — Leaf growth parameters under thermal stress. Graph representation of leaf growth parameters of two populations of Brassica oleracea under thermal stress conditions. Simple linear regression curves of each temperature are represented. [file 12870_2015_535_MOESM1_ESM.pdf]

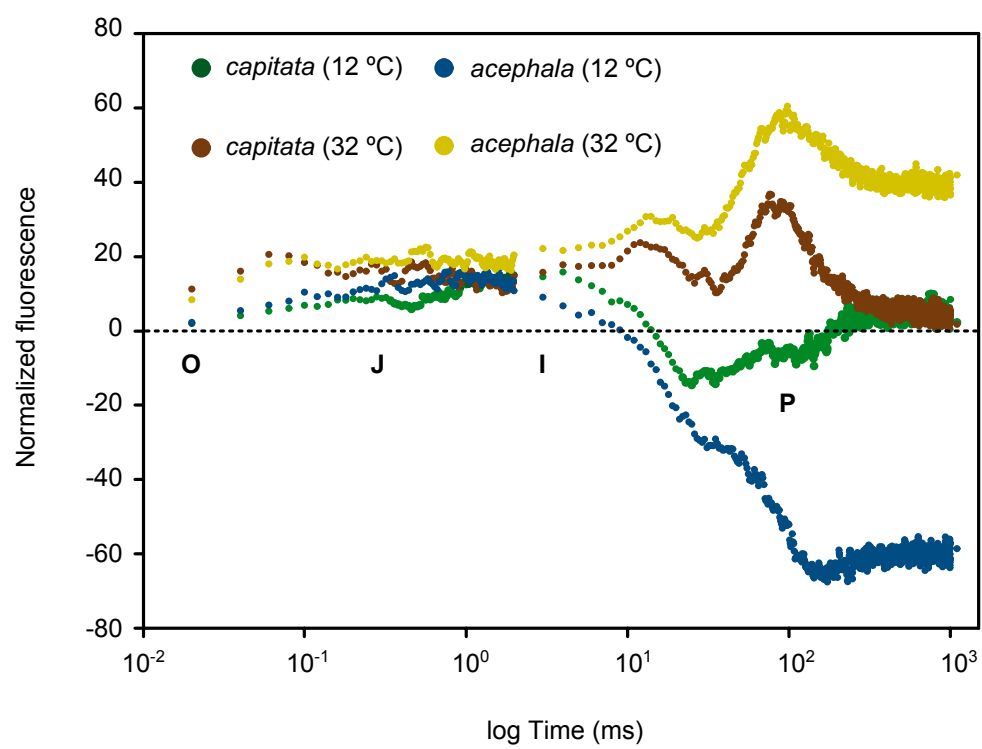

Supplement: Additional file 2: Figure S2. — Normalized chlorophyll a fluorescence transient curve. Chlorophyll a fluorescence transient curve. Data from the control were used to normalize the curve. [file 12870_2015_535_MOESM2_ESM.pdf]
