# Supplementary material for: Effect of temperature stress on the early vegetative development of Brassica oleracea L
Source: BMC Plant Biol. 2015 Jun 16;15:145. doi: 10.1186/s12870-015-0535-0 (PMC4467057; doi:10.1186/s12870-015-0535-0)
Supplement: Additional file 3: Table S1. — List of parameters of the JIP-test. Summary of parameters and formula description using data extracted from the OJIP transient. [file 12870_2015_535_MOESM3_ESM.docx]

| **Fluorescence**  **parameter** | **Formula** |
| --- | --- |
| F_0_ | ≈ F_50µs_ |
| M_0_ | $=4\times\left(\frac{F_{300\mu s}-F_{0}}{F_{m}-F_{0}}\right)$ |
| S_m_ | $=\frac{Area}{F_{v}}$ |
| $\phi_{P_{0}}$ | $=\frac{F_{v}}{F_{m}}$ |
| PI_ABS_ | $=\left(\frac{RC}{ABS}\right)\times\left(\frac{\phi_{P_{0}}}{1-\phi_{P_{0}}}\right)\times\left(\frac{\psi_{0}}{1-\psi_{0}}\right)$ |
| ABS/RC | $=M_{0}\times\left(\frac{1}{V_{J}}\right)\times\left(\frac{1}{\phi_{PO}}\right)$ |
| TR_0_/RC | $=\frac{M_{0}}{V_{J}}$ |
| ET_0_/RC | $=\left(\frac{M_{0}}{V_{J}}\right)\times\left(1-V_{J}\right)$ |
| DI_0_/RC | = ABS/RC – TR_0_/RC |

**Additional Table 1.** Summary of parameters and formula description using data extracted from the OJIP transient.
